# Ensemble learning driven Kolmogorov-Arnold Networks-based Lung Cancer classification

**DOI:** 10.1371/journal.pone.0313386

**Published:** 2024-12-31

**Authors:** Abdul Rahaman Wahab Sait, Eid AlBalawi, Ramprasad Nagaraj

**Affiliations:** 1 Department of Archives and Communication, King Faisal University, Hofuf, Kingdom of Saudi Arabia; 2 Department of Computer Science, College of Computer Science and Information Technology, King Faisal University, Al Hofuf, Kingdom of Saudi Arabia; 3 Department of Biochemistry, S S Hospital, S S Institute of Medical Sciences & Research Centre, Rajiv Gandhi University of Health Sciences, Davangere, Karnataka, India; Queensland University of Technology, AUSTRALIA

## Abstract

Early Lung Cancer (LC) detection is essential for reducing the global mortality rate. The limitations of traditional diagnostic techniques cause challenges in identifying LC using medical imaging data. In this study, we aim to develop a robust LC detection model. Positron Emission Tomography / Computed Tomography (PET / CT) images are utilized to comprehend the metabolic and anatomical data, leading to optimal LC diagnosis. In order to extract multiple LC features, we enhance MobileNet V3 and LeViT models. The weighted sum feature fusion technique is used to generate unique LC features. The extracted features are classified using spline functions, including linear, cubic, and B-spline of Kolmogorov–Arnold Networks (KANs). We ensemble the outcomes using the soft-voting approach. The model is generalized using the Lung—PET–CT–DX dataset. Five–fold cross-validation is used to evaluate the model. The proposed LC detection model achieves an impressive accuracy of 99.0% with a minimal loss of 0.07. In addition, limited resources are required to classify PET / CT images. The high performance underscores the potential of the proposed LC detection model in providing valuable and optimal results. The study findings can significantly improve clinical practice by presenting sophisticated and interpretable outcomes. The proposed model can be enhanced by integrating advanced feature fusion techniques.

## 1. Introduction

In terms of global mortality rates, LC remains one of the most prevalent factors [1, 2]. There has been an exponential growth in incidence and mortality during the past few decades [2, 3]. LC rates have increased by approximately three percent in the past two decades [1, 2, 4]. Recent studies [1], [3, 5] highlight the significance of chest radiographs, computed tomography, magnetic resonance imaging, and positron emission tomography / computed tomography (PET / CT) in identifying LC. Chest radiograph is the primary diagnostic tool for detecting lung conditions. The accuracy of abnormality detection using chest radiographs depends on the radiologist’s expertise. In addition, an insignificant quantity of ionizing radiation is required to generate an image of the chest’s interiors [5–7]. It can aid in diagnosing conditions including shortness of breath, persistent cough, fever, chest discomfort, and damage to the chest wall and lungs [8]. The limited resolution of chest radiographs makes it challenging to identify tiny lung nodules or early-stage malignancies, especially those under 1 centimeter [9–11]. Chest radiographs fail to stage disease spread, essential for therapy and prognosis [12]. They are less effective in LC management, where exact staging is crucial.

In clinical settings, PET / CT images are frequently used to diagnose various chest conditions. PET/CT is preferable to other imaging techniques for LC detection and diagnosis [13]. The metabolic insights of PET and the anatomical detail of CT are incorporated with this hybrid imaging approach, which enhances the early detection of LC. CT scans reliably identify lung lesions’ size, shape, and location, enabling anatomical mapping. Visualizing tissue and organ anatomy assists in identifying lung nodules and masses. This approach is user-friendly, and the operation exposes patients to minimal radiation [14]. It can reveal abnormalities in the heart, lungs, blood vessels, and bronchial tubes. Magnetic resonance imaging is highly beneficial in producing detailed images of soft tissues. However, it may overlook the key indicators of LC. PET/CT is better at staging LC as it visualizes tumor metabolism and anatomy. It can identify metastases more effectively than magnetic resonance imaging.

According to recent studies [3, 15, 16], the use of PET / CT in LC screening may increase the rate of survival. Computer vision technologies have been used to automatically detect lung abnormalities using PET/CT images [17, 18]. Due to recent advances in processing power and the availability of massive annotated datasets, deep learning (DL) has become the dominant method in medical imaging. DL classifiers facilitate artificial intelligence (AI)–based disease diagnosis [19]. These models offer an effective platform to aid medical professionals in examining and detecting malignant tumors at earlier stages [20, 21]. Convolutional neural network (CNN) supports healthcare practitioners in detecting chest diseases and cancer identification from complex medical images. The transfer learning technique is part of the DL techniques used to analyze medical images and solve complex real-time problems. DL models based on transfer learning applications achieved optimal accuracy in classifying medical images [21]. Pre-trained models apply prior expertise to address challenging problems [22].

DL applications for medical image classification empower clinicians and enable fast clinical decision-making [22]. The training dataset plays a significant role in improving the performance of DL-based medical image classification systems. One of the critical challenges of DL in medical image analysis is the inconsistency of the healthcare center’s ability to deliver adequate medical images for training sets [23, 24]. Dataset bias occurs when the available data is inaccurate or misleading the actual state of a medical condition [24]. Due to the data mismatch, benchmark algorithms may perform negatively in real time. Dataset bias may negatively influence the variety of medical imaging modalities, including PET / CT images, images of the retina and brain, images of tissue sections, and skin biopsies [24].

There is a significant improvement in the development of AI-based preventative medicine, diagnostic support, and personalized medicine. In recent decades, significant collaborative cancer initiatives have resulted in the development of various clinical, medical imaging, and sequencing databases [25]. These databases enable researchers to enhance LC detection, treatment, and clinical outcomes. However, a significant amount of time and expertise is required for drawing insight from the high-dimensional data types. The rapidly expanding cancer-associated databases present a significant challenge in developing an LC detection model [25].

The existing LC detection techniques are limited in offering a practical decision to clinicians and radiologists. The shortcomings of the feature extraction techniques reduce the performance of the LC detection models. The success of DL-based disease diagnosis applications depends on the availability of patient data. The generalization of these applications is necessary in order to deploy them in healthcare centers and provide early diagnosis of LC. Ensemble learning has recently been widely applied to enhance prediction model accuracy and resilience across multiple areas. It uses different frameworks to produce an integrated model, incorporating their strengths to improve performance and generalizability. In addition, it may enhance LC detection and diagnosis accuracy and reliability over single-model methods. These features of ensemble learning motivate us to build an LC detection model using it. The healthcare centers demand a practical LC detection framework with limited resources. Thus, this study develops a framework for detecting LC from PET / CT images. The contributions of the study are

1. Implementation of a compelling feature extraction technique for building an LC classifier using the PET / CT images,
2. Developing an ensemble learning-driven Kolmogorov Arnold Networks (KANs)-based LC classification,
3. Evaluation of the proposed LC detection model by comparing it with the baseline models using the benchmark metrics.

The remaining parts of the study are structured as follows: The related works are presented in Section 2. Section 3 outlines the research methodology of the LC detection framework. It presents detailed information on image acquisition, pre-processing, feature selection, and classification. The experimental outcomes of the LC detection framework is discussed in section 4. The significance of the proposed study is discussed in section 5. Lastly, section 6 concludes the study with the limitations and future directions.

## 2. Related works

Feature extraction is the process of identifying the crucial patterns associated with disease or conditions approach [25]. CNNs may identify LC characteristics such as nodules, masses, and abnormal tissue patterns. To identify patterns, the input image is processed through a series of convolutional layers with [25] several filters that slide over the image. These filters identify early-layer edges, textures, and gradients. The CNN model uses deeper layers to capture forms, structures, and increasingly complicated patterns that indicate particular anatomical structures or clinical disorders. The pre-trained CNN models, including VGG, ResNet, and Inception, were employed for LC classification. The existing studies revealed the significant role of the pre-trained CNN models in extracting the crucial patterns of LC using multiple imaging modalities. Diagnostic predictions are made by integrating these high-level features in the CNN’s final layers with an activation function [25]. The ability of CNNs to automatically and efficiently extract key information from medical images allows for diagnosing various diseases in the earlier stages. CNNs leverage hierarchical layers to extract features from medical images across multiple abstraction levels.

Park et al. (2021) [25] developed an LC detection model using ResNet-18 architecture. They trained the model with CT images of 359 individuals. The model was evaluated using five-fold cross-validation. It achieved a considerable area under the receiving operating characteristic curve (AUROC) value of 83.7. The findings reveal the significance of CT features in enhancing prediction accuracy.

Sepehri et al. (2021) [26] compared the abilities of ML models in detecting LC. A total of 138 individuals’ PET / CT images were used to train the models. The authors employed a voting approach to compute the results. The findings highlighted that different models utilized unique features of PET / CT in identifying LC.

Chen et al. (2022) [27] proposed a stacked DL model for LC detection. They used ResNet models as base predictors. The Support Vector Machine (SVM) model was used as a meta-learner to generate an outcome. The findings of the experimental analysis revealed that the stacked model outperformed the individual ResNet variants.

Huang et al. (2022) [28] developed a machine-learning model using PET / CT images to identify lung conditions. They employed a pre-trained CNN model for the feature extraction. Random Forest classifier was used to classify the features and compute the malignancy progression. The ensemble model achieved an accuracy of 66.4% with an AUROC of 0.65.

Sengodan et al. (2023) [29] introduced a model to detect LC in the earlier stages. They employed an ensemble learning approach using PET / CT images to predict LC. They employed the multi-populational neighborhood particle swarm optimized modified ensemble fast learning approach to improve the prediction accuracy. The model was trained using the lung image database consortium image–image database resource initiative (LTDC-IDRI) dataset that contains 1018 thoracic CT images.

Onozato et al. (2023) [30] developed multiple machine-learning models to predict the pathological invasiveness of LC using PET / CT images. They ensembled seven models to enhance the prediction accuracy. They evaluated the model using K-fold cross-validation. The model achieved an AUROC of 88.0 with an accuracy of 80.4%.

Sultana et al.(2024) [31] proposed an ensemble learning model for LC detection. They used MobileNet V2, VGG19, and ResNet 50 models to extract the features from the CT images. The weighted average ensemble technique was used to classify the extracted features. The model obtained an overall accuracy of 98.93%. However, it required substantial computational resources to make a final decision. The characteristics of the existing LC models are presented in Table 1.

**Table 1:** Features and limitations of the existing models.

Authors	Methodology	Dataset (No. of Individuals)	Performance	Limitations
Park et al. (2021) [25]	ResNet-18-based transfer learning approach	359	Accuracy = 87.3% AUROC = 0.837 AUPRC = 0.84	Demand substantial training and huge computational resources.
Sepehri et al. (2021) [26]	Feature fusion approach	138	Accuracy = 93.6% AUROC = 0.89 AUPRC = 0.81	Focused on binary classification. The performance may vary in the multi-class classification.
Chen et al. (2022) [27]	High level SVM	147	Accuracy = 80.9% AUROC = 0.87 AUPRC = 0.85 Sensitivity = 85.0% Specificity = 76.0%	The SVM model may struggle with large datasets, affecting the overall prediction accuracy.
Huang et al. (2022) [28]	Pre-trained CNN with Random Forest	965	Accuracy = 66.4% AUROC = 0.66 AUPRC = 0.65	Fine-tuning the pre-trained model can lead to overfitting, reducing the model’s generalization accuracy.
Sengodan et al. (2023) [29]	Ensemble SVM	1018	Accuracy = 97.1% AUROC = 0.89 AUPRC = 0.87	The effectiveness of the ensemble SVM depends on the choice of kernel function, which may cause challenges in the optimization process.
Onozato et al. (2023) [30]	Ensemble learning approach	873	Accuracy = 80.4% AUROC = 0.88 AUPRC = 0.86	Training and evaluating multiple machine learning models demand huge computational resources that may cause challenges in the resource–constrained environment.
Sultana et al.(2024) [31]	Weighted average ensemble technique	147	Accuracy = 98.9% AUROC = 0.9 AUPRC = 0.87	Focused on binary classification. Sub optimal weights may result in poor model performance. In addition, substantial computational resources are required to achieve an optimal outcome.

The existing studies outlined that the pre-trained models outperformed the traditional feature extraction models in terms of speed and accuracy. The performance metrics, including accuracy, precision, recall, F1-score, Mathew’s correlation coefficient (MCC), Cohen’s Kappa, AUROC, and area under the precision-recall curve (AUPRC), were used to evaluate the performance of LC detection models. Computational costs are critical for deploying LC detection models in resource-constrained settings. Generalization is a model’s capacity to perform effectively on data from a different distribution than the training data. Medical imaging Models are frequently employed across multiple entities and patient demographics. Single-source LC detection models may not generalize well to various situations due to variances in image acquisition, scanner types, and patient demographics. Ensemble learning approaches may offer robust and optimal LC detection models. These approaches can mitigate overfitting challenges. The model generalization can be achieved by leveraging the strength of multiple models. In image analysis, DL models are typically viewed as black boxes. Multiple layers of non-linear transformations make it challenging to understand the prediction process. To make informed therapeutic decisions, determining a prediction’s logic is essential for gaining healthcare professionals’ confidence. Healthcare professionals may trust explainable medical diagnostic models. For instance, they can utilize the nodule characteristics, including size, shape, and texture, to comprehend the LC model’s outcome. These characteristics assist in validating the diagnosis, identifying inaccuracies, and guiding medical investigations.

## 3. Materials and methods

**Fig 1:**
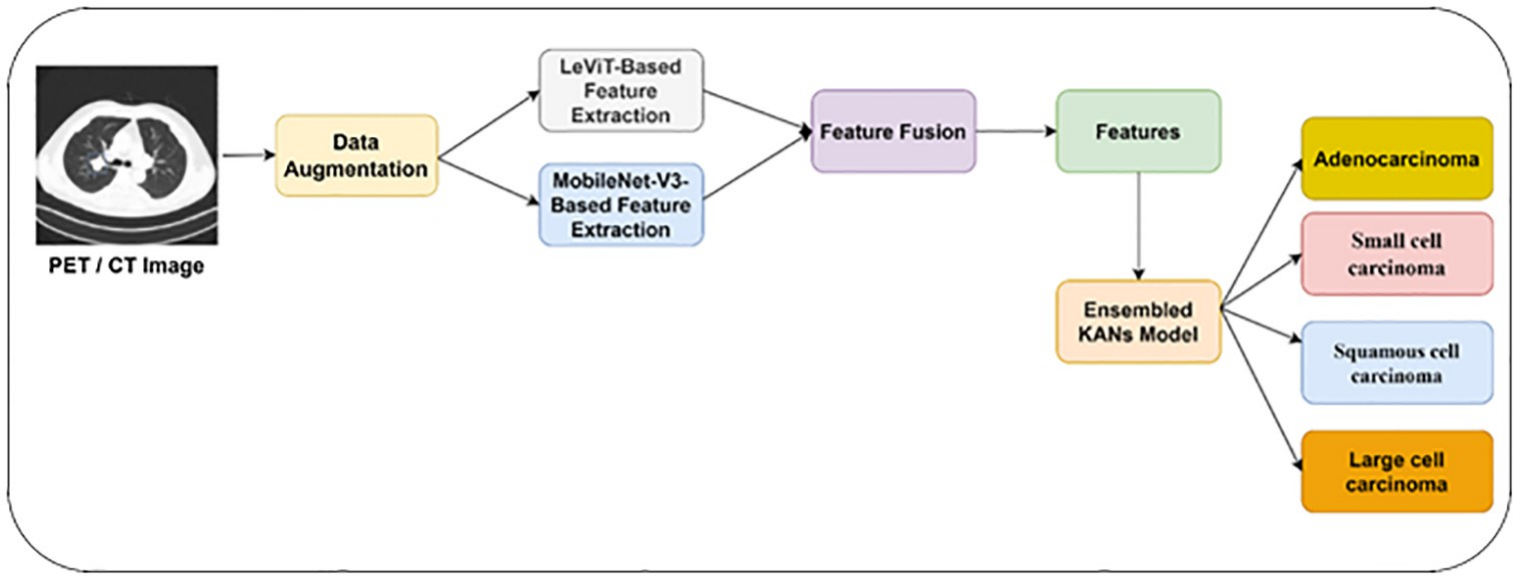
Proposed LC detection model.

We propose a model for identifying LC using ensemble learning techniques. Fig 1 offers the proposed methodology for classifying PET / CT images into multiple classes. We employ MobileNet V3 and LeViT models to extract diverse features of LC. They apply data augmentation techniques to train the feature extraction models. A feature fusion technique is used to identify the unique LC features. The extracted features are classified using an ensembled KANs model.

### 3.1 Data acquisition

**Fig 2:**
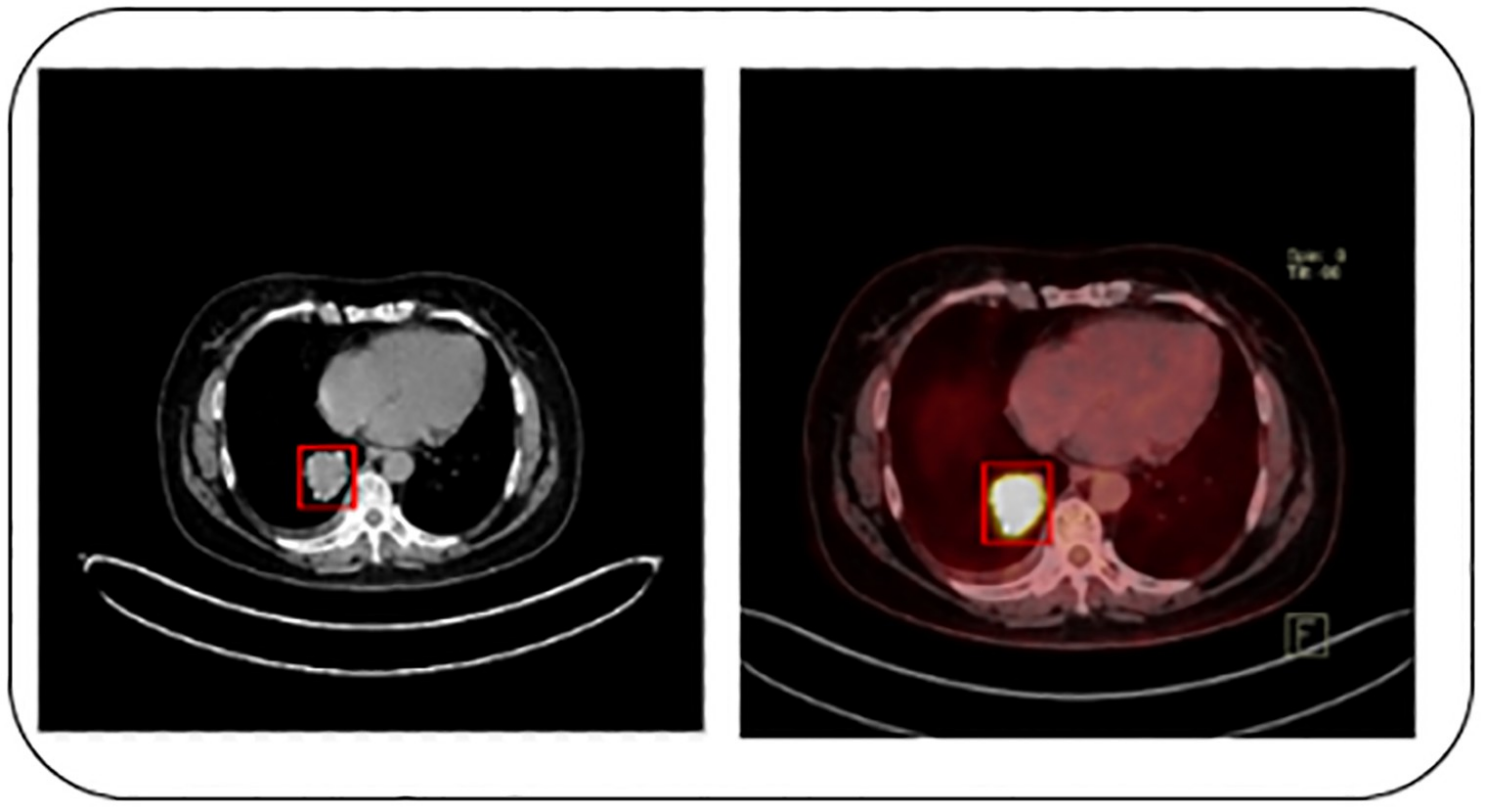
Sample PET / CT images.

The Lung—PET–CT–DX dataset [32] is used to train and test the proposed model. It contains LC types, including Adenocarcinoma (20894 images), Small cell carcinoma (3116 images), Large cell carcinoma (201 images), and Squamous cell carcinoma (7351 images). The CT image resolution is 512 × 512 pixels, and PET image resolution is 200 × 200 pixels. The larger number of images enhances the performance of the DL models. Therefore, we trained the model using the classes including, Adenocarcinoma, Small cell carcinoma, Large cell carcinoma, and Squamous cell carcinoma images in this study. In addition, they applied data augmentation techniques to increase the dataset size. Fig 2 presents the sample images of the dataset.

### 3.2 Feature extraction

**Fig 3:**
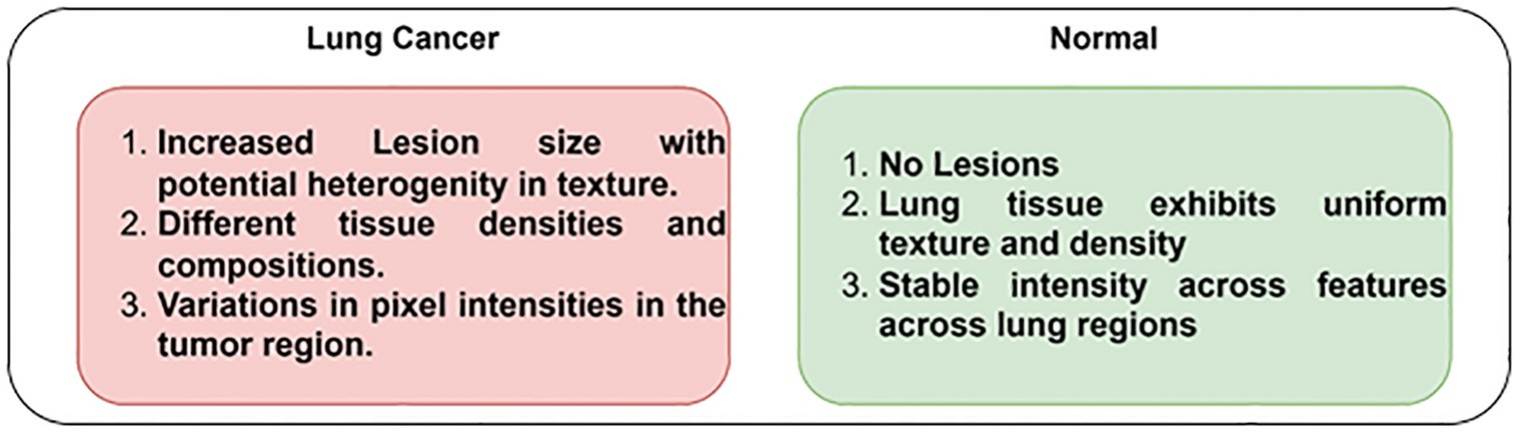
Significant features of Lung Cancer and normal PET / CT images.

Extracting the features from the PET / CT images plays a crucial role in detecting and diagnosing LC. The features, including nodule size, shape, and texture, enable the proposed LC detection model to uncover intricate patterns associated with LC. The diverse set of features enhances the potential of the proposed model in differentiating normal and LC individuals. The high-quality features can significantly improve the model’s prediction accuracy, leading to better treatment outcomes. Fig 3 shows the significant features of PET / CT images, differentiating normal and LC individuals.

#### 3.2.1 MobileNet V3-based feature extraction

MobileNet V3 model can capture low-level features and progressively generate high-level features associated with LC. The efficient architecture motivates us to employ the MobileNet V3 model to build the proposed LC detection model with limited computational resources. The h-Swish activation function supports the proposed model in balancing computational power and accuracy trade-offs. To extract LC features, we employ weights of MobileNet V3 model. In order to improve the efficiency of the MobileNet V3-based feature extraction, we use pruning and early stopping strategies. The pruning strategy identifies and removes redundant channels with minimal impact on the model’s performance. Magnitude-based pruning is used to analyze the weights of each channel. We remove channels with smaller weight magnitudes across the entire layer of the feature extraction model. Multiple layers are used to extract features. The depthwise convolution layer processes individual channels in order to extract spatial features. The subsequent pointwise convolution assigns weights to the features. The weights in the pointwise convolution are used to determine the importance of the extracted features. After the successful pruning, we fine-tuned the model with Adam optimization to guarantee the model adaptation to the reduced complexity. By integrating an early stopping strategy with pruning, we maintain the model size and performance. The mathematical form of the recommended MobileNet V3-based feature extraction is presented in Eq 1.

$$F_{MN}=MobileNet\;V3\left(CT,ES,MP\right)$$ (1)

where *F*_*MN*_ is the features extracted using MobileNet V3 model, CT is the CT image, ES is the early stopping strategy, and MP is the magnitude-based pruning.

#### 3.2.2 LeViT-based feature extraction

LeViT can capture the intricate patterns of LC from the CT images using convolutional layers and transformer blocks. The integration of convolutional layers minimizes the computation time of LeViT. Unlike the existing vision transformers, LeViT offers faster inference, leading to optimal outcomes with limited computational resources. We are motivated by the LeViT feature extraction ability. However, they improve the LeViT performance by employing an iterative pruning strategy. Using this strategy, a small percentage of channels is pruned at a time. Subsequently, the model is retrained, and the accuracy drop is evaluated. A controlled model size reduction is achieved by implementing an early stopping strategy with iterative pruning. LeViT’s attention layer dynamically assigns weights to features based on their relevance to the classification process. We employ Hyberband optimization to fine-tune the LeViT-based feature extraction. Eq 2 outlines the computational form of the suggested feature extraction.

$$F_{LeViT}=LeViT\left(CT,IP,Hb\right)$$ (2)

where *F*_*LeViT*_ is the LC feature, *CT* is the image, *IP* is the iterative pruning, and *Hb* is the Hyberband optimization.

### 3.3 KANs-based LC detection

The kolmogrov-Arnold Networks (KANs) model is a variant of artificial intelligence architecture that has the potential to achieve high accuracy and interpretability in a real-time setting [33]. It uses a univariate function called a spline to determine the importance of the feature. A spline is a learnable one-dimensional function that controls the residual attention function. The KANs architecture can support the proposed model in computing the outcomes with few computational resources. In this study, we employ KANs in order to classify the CT image features into multiple classes. The mathematical expression of KANs is expressed in Eq 4.

$$F\left(x\right)=\sum_{K=1}^{2n+1}\theta_{K}\left(\sum_{P=1}^{n}\theta_{K,P}\left(x_{p}\right)\right)$$ (3)

where *θ*_*K*_ and *θ*_*K*,*P*_ are the univariate functions using the parameters *K* and *P*, *x*_*p*_ is the number of parameters, and n is the total number of features.

**Fig 4:**
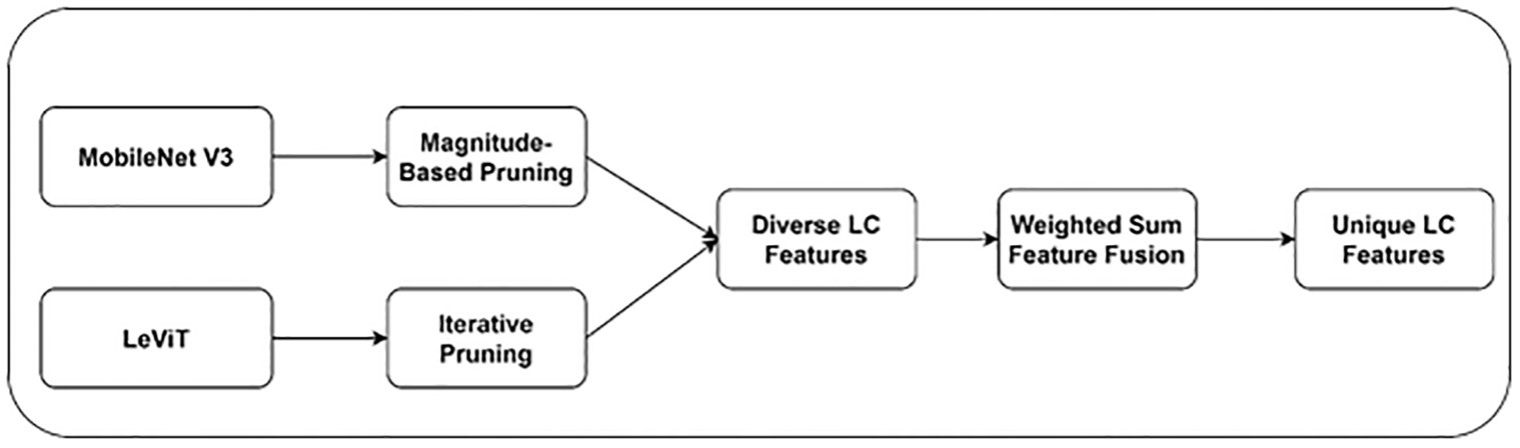
Recommended feature fusion technique.

LeViT and MobileNet V3 models capture multiple features associated with LC. Combining these features can generate a broader spectrum of LC features. In addition, feature fusion can boost the LC model’s performance. Fig 4 outlines the recommended feature fusion approach. We employ a weighted-sum fusion technique to select the crucial features. They assign weights to the extracted features.

The mathematical form of the weighted sum of features is presented in Eq 3.

$$F_{fused}=\sum_{n=1}^{m}W_{m}\times F_{m}$$ (4)

where *F*_*fused*_ is the fused feature, *W*_*m*_ is the weight, *F*_*m*_ is the individual features, and m is the total number of features.

After computing the fused feature vector, the individual features are ranked based on their weights. The features with high weightage indicate higher importance. Recursive feature elimination retains the most informative features according to their rankings. The selected features are passed to the subsequent model training and evaluation processes.

During the training phase, the fine-tuned KANs model automatically determines the key features based on the classification accuracy. In order to achieve an optimal outcome, we followed the ensemble learning approach by integrating the outcomes through three spline types, including Cubic, B-Spline, and Linear. They employ a soft voting approach to generate the final outcome.

The concept of a probability distribution over the classes in the soft voting approach assists the proposed LC detection model in making a decision with optimal accuracy. We train the model with three spline types. Each spline type can capture unique features of LC from the CT images. Each base model generates a probability distribution for each LC class. An average probability is calculated for each class across all models. The final prediction is made by choosing the class with the highest probability. The proposed ensembled approach mitigates individual biases and variances by aggregating the predictions of multiple spline types. We employ the Hyperband optimization algorithm to fine-tune the KANs model. Hyperband is an adaptive resource allocation and early-stopping approach-based optimization algorithm. It effectively allocates resources to different hyperparameter configurations. The hyperparameters, including learning rate, number of layers, activation functions, batch size, weight initialization, and dropout rates, are fine-tuned using the Hyperband optimization. The algorithm randomly selects the KANs hyperparameters using different learning rates, number of layers, and batch sizes. It maintains a trade-off between different hyperparameters and refines the best-performing configurations.

To understand the behavior of the proposed model, we integrate local interpretable model-agnostic explanations (LIME) with KANs-based LC detection model. The computational form of the LIME integration is presented in Eq 5.

$$F_{P\;}=KANs.Lime\left(X_{train},FN,CN,discrete\;continuous=true\right)$$ (5)

where *F*_*P*_ is the final prediction, *X*_*train*_ is the train set, *FN* is the feature, *CN* is the class label, and “.” represents the sub function (Lime) of KANs model.

The quantization technique reduces the computational cost by converting high-precision numbers to lower-precision numbers. It supports the proposed model’s generation of results with limited resources.

## 4. Results

The proposed LC detection model is implemented using Python 3.8.0, Windows 10, 8 GB RAM, and NVIDIA Titan RTX GPU. TensorFlow and Keras libraries are used to improve the images. The benchmark metrics are used to evaluate the performance of the proposed LC detection framework. Accuracy is used to measure the prediction level of the LC detection models. Recall, precision, and F1-measure are employed to guarantee the framework’s ability to determine true positive, true negative, false positive, and false negative images. In addition, MCC and Kappa are used to evaluate the framework’s classification ability. The Monte Carlo dropout method is used to compute the prediction uncertainty of the proposed framework. The proposed framework is executed multiple times in order to identify its capability to deal with overfitting challenges.

**Fig 5:**
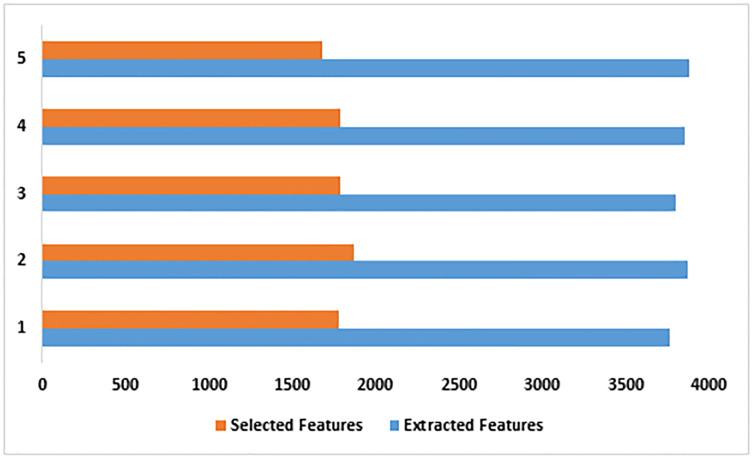
Fold-wise extracted and selected features.

The proposed framework achieved a better outcome in the 34^th^ epoch. However, the researchers extended the experiment to the 43^rd^ epoch. There is no significant difference in the outcome. Thus, the 43^rd^ epoch is considered the topmost epoch for generating high outcomes. Fig 5 highlights the average number of extracted and selected features in each fold. The recommended feature fusion technique selected the crucial patterns associated with LC classes.

**Fig 6:**
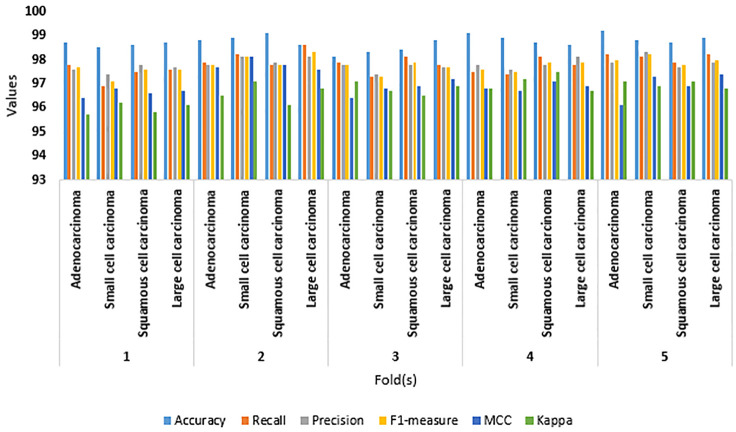
Outcomes of performance analysis.

Table 2 reveals the outcome of the five-fold cross-validation. The proposed framework obtained an average accuracy, recall, precision, F1-measure, MCC, and Kappa of 99.0, 97.8, 97.8, 97.8, 97.0, and 96.7, respectively. The performance of the proposed LC detection framework in each fold is presented in Fig 6.

**Table 2:** Findings of performance evaluation.

Fold (s)	Classes	Accuracy	Recall	Precision	F1-measure	MCC	Kappa
1	Adenocarcinoma	99.2	97.8	97.6	97.7	96.4	95.7
	Small cell carcinoma	99.1	96.9	97.4	97.1	96.8	96.2
	Squamous cell carcinoma	98.9	97.5	97.8	97.6	96.6	95.8
	Large cell carcinoma	98.8	97.6	97.7	97.6	96.7	96.1
2	Adenocarcinoma	99.2	97.9	97.8	97.8	97.7	96.5
	Small cell carcinoma	98.9	98.2	98.1	98.1	98.1	97.1
	Squamous cell carcinoma	99.1	97.8	97.9	97.8	97.8	96.1
	Large cell carcinoma	98.6	98.6	98.1	98.3	97.6	96.8
3	Adenocarcinoma	99.5	97.9	97.8	97.8	96.4	97.1
	Small cell carcinoma	99.4	97.3	97.4	97.3	96.8	96.7
	Squamous cell carcinoma	99.3	98.1	97.8	97.9	96.9	96.5
	Large cell carcinoma	98.8	97.8	97.7	97.7	97.2	96.9
4	Adenocarcinoma	99.1	97.5	97.8	97.6	96.8	96.8
	Small cell carcinoma	98.9	97.4	97.6	97.5	96.7	97.2
	Squamous cell carcinoma	98.7	98.1	97.8	97.9	97.1	97.5
	Large cell carcinoma	98.6	97.8	98.1	97.9	96.9	96.7
5	Adenocarcinoma	99.2	98.2	97.9	98.0	96.1	97.1
	Small cell carcinoma	98.8	98.1	98.3	98.2	97.3	96.9
	Squamous cell carcinoma	98.7	97.9	97.7	97.8	96.9	97.1
	Large cell carcinoma	98.9	98.2	97.9	98.0	97.4	96.8
Average		99.0	97.8	97.8	97.8	97.0	96.7

**Fig 7:**
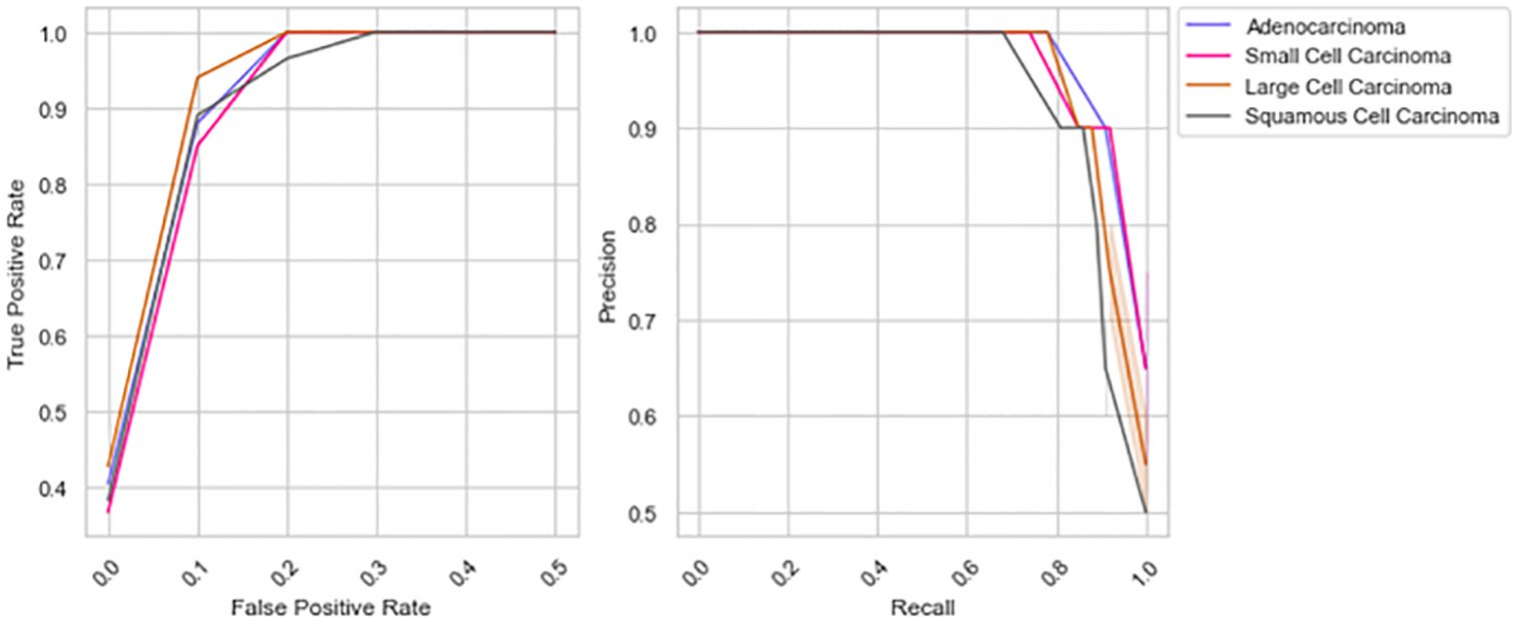
Outcomes of the AUROC and AUPRC analysis.

Fig 7 highlights the classification ability of the proposed model. The recommended feature extraction and classification techniques addressed the challenges in classifying LC using PET / CT images. The higher AUROC and AUPRC reflect the model’s capability to differentiate the LC classes.

Table 3 outlines the prediction uncertainty of the proposed framework. To measure the prediction uncertainty, the researchers computed a confidence interval (CI) of 95%, a standard deviation (SD), and Entropy. The findings show that the proposed framework can generate an effective result from low-resolution images. In addition, the shortcomings of the PET / CT images and pre-trained CNN model, including overfitting and vanishing gradient, were addressed using the data augmentation techniques.

**Table 3:** Outcomes of statistical analysis.

Fold (s)	CI @95%	SD	Entropy
1	[95.4–96.7]	0.0007	0.0031
2	[95.7–96.8]	0.0009	0.0175
3	[96.1–97.3]	0.0014	0.0215
4	[96.7–97.8]	0.0019	0.0218
5	[96.9–97.4]	0.0017	0.0179

**Fig 8:**
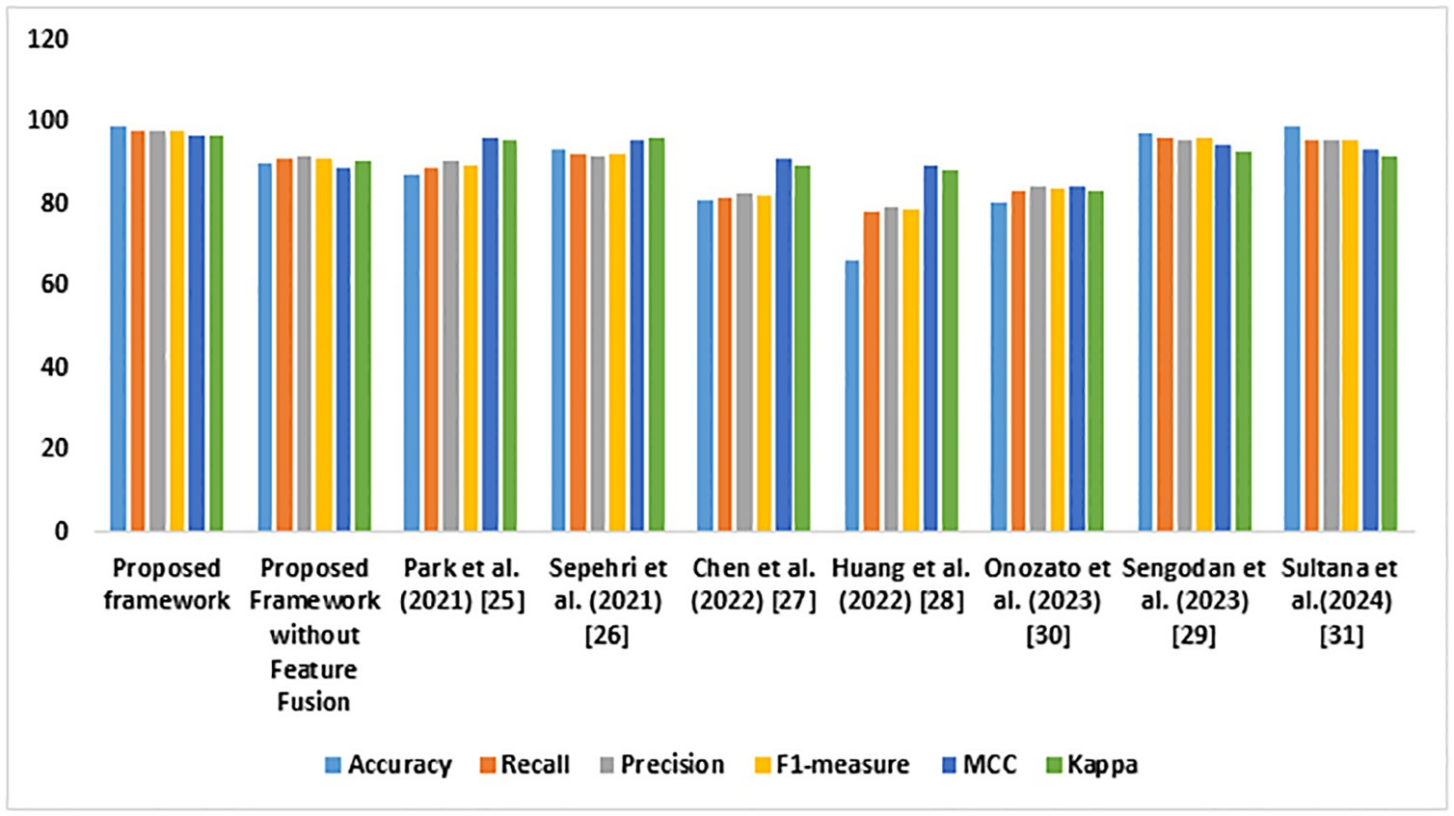
Comparative analysis outcome.

Table 4 presents the experimental outcome of the comparative analysis. The proposed framework obtained a superior outcome than the baseline models. BOHB hyper-parameter optimization fine-tuned the proposed model’s performance. The high F1 Measure highlights the significance of the recommended framework in processing the images. Fig 8 illustrates the performance of the LC detection frameworks.

**Table 4:** Comparative analysis outcomes.

Models / Metrics	Accuracy	Recall	Precision	F1-measure	MCC	Kappa
Proposed framework	99.0	97.8	97.8	97.8	97.0	96.7
Proposed Framework without Feature Fusion	90.2	91.4	91.5	91.4	89.1	90.4
Park et al. (2021) [25]	87.3	88.9	90.5	89.6	96.1	95.8
Sepehri et al. (2021) [26]	93.6	92.4	91.8	92.1	95.8	96.1
Chen et al. (2022) [27]	80.9	81.6	82.5	82.0	90.9	89.4
Huang et al. (2022) [28]	66.4	78.4	79.5	78.9	89.4	88.4
Onozato et al. (2023) [30]	80.4	83.4	84.6	84.0	84.1	83.5
Sengodan et al. (2023) [29]	97.1	96.4	95.9	96.1	94.3	92.8
Sultana et al.(2024) [31]	98.9	95.6	95.8	95.7	93.4	91.9

Table 5 outlines an individual framework’s computational parameters and learning rates for classifying normal and abnormal images. The proposed framework obtained less computational loss by ensembling the different spline functions. MobileNet V3 and LeViT extracted the critical features associated with LC with limited computational power. Thus, the proposed LC detection framework required fewer parameters compared to the existing frameworks.

**Table 5:** Computational strategies.

Frameworks / Metrics	Parameters (Millions(M))	FLOPs (Giga (G))	Learning Rate	Loss
Proposed framework	19	27	0.0004	0.07
Park et al. (2021) [25]	31	39	0.0006	0.13
Sepehri et al. (2021) [26]	39	42	0.0006	0.21
Chen et al. (2022) [27]	29	39	0.0004	0.42
Huang et al. (2022) [28]	33	38	0.0004	0.37
Onozato et al. (2023) [30]	41	37	0.0008	0.41
Sengodan et al. (2023) [29]	26	34	0.0006	0.32
Sultana et al.(2024) [31]	29	33	0.0006	0.43

**Fig 9:**
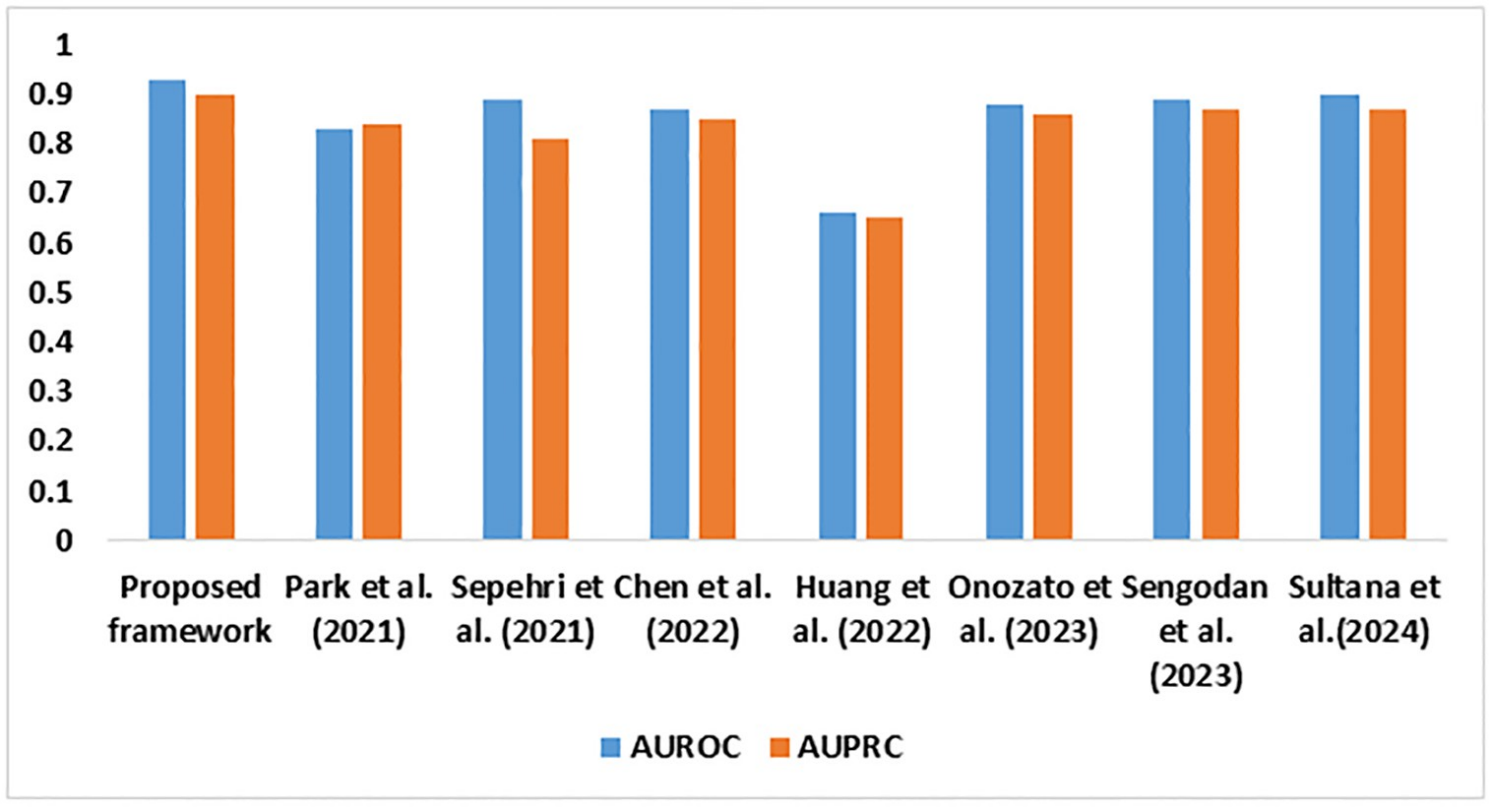
The findings of AUROC and AUPRC analysis.

Fig 9 presents the superior performance of the proposed model in managing class imbalances. AUROC and AUPRC are crucial in validating the capability of AI-based medical applications. The higher AUROC of 0.93 outlines the enhanced ability of the proposed model to differentiate the LC types. The existing models struggle to identify the key indicators of LC, leading to lower AUROC. Similarly, the higher AUPRC of 0.91 highlights the robustness of the proposed LC detection model in detecting positive LC cases. It is evident that the proposed model is reliable in clinical practice, identifying LC with fewer false positives and negatives.

## 5. Discussions

The proposed LC detection framework achieved an optimal outcome with limited resources. It outperformed the recent LC detection approaches. The significance of the study lies in its ability to improve the diagnosis of LC using effective feature fusion and ensemble learning techniques. By leveraging the potential of MobileNet V3 and LeViT, and multiple spline functions, the proposed LC detection model outperformed the state-of-the-art models. We reduced the false positives and negatives by enabling the feature importance functionalities of LeViT and MobileNet V3 models. Integrating PET / CT improved the model’s potential to detect small and early-stage lesions. LIME provides model interpretability by correlating the model’s predictions. This capability can assist clinicians in comprehending the logic of the proposed LC model prediction. LIME highlights the crucial features, including textures, shapes, or regions of CT images, that influenced the decisions of the proposed model. It builds trust in the proposed model, ensuring accuracy and explainable predictions.

The findings revealed that the proposed model addressed the challenges in processing PET / CT images and generated superior outcomes than the existing LC detection frameworks. Table 2 highlights the prediction uncertainties of the proposed framework. It is evident that the proposed LC detection model achieved a CI of 96.8–98.2 with an SD of 0.0007. The suggested approach can potentially improve accuracy in characterizing and classifying lung nodules over existing techniques. The classification accuracy is improved by combining feature selection and a pre-trained network. The suggested framework achieves a more excellent detection regardless of the nodule size.

Park et al.(2021) [25] used the ResNet 18 model for feature extraction. The ResNet 18 architecture is sensitive to hyperparameters, including learning rate, batch size, and weight decay. Identifying an optimal set of hyperparameters is challenging in the context of LC detection. In contrast, the recommended LeViT and MobileNet V3 models produced unique LC features with limited parameters and FLOPs.

Sengoden et al. (2021) [26] employed the particle swarm optimization architecture in classifying LC images. The risk of premature convergence, increased memory requirements, and implementation complexity reduced the efficiency of the Sengoden et al. model. On the other hand, integrating the quantization technique minimized the implementation complexity of the proposed LC detection model.

The weighted average ensemble learning approach supported the Sultan et al. model in obtaining considerable accuracy. However, the challenges in weight selection, such as interpretability issues and the risk of overfitting, can reduce the model’s generalization capability. Similarly, the black box nature of the pre-trained models, including MobileNet V2, VGG19, and ResNet 50, offered limited model’s interpretability to the Sultana et al. [31], Chen et al. [27], Huang et al. [28], Onazato et al. [30] models. In addition, the limited functionality of the SVM model caused challenges to Chen et al., model in classifying PET / CT images. The lack of effective feature extraction reduced the prediction accuracy of the Sepehri et al. model.

Despite the promising potential of the proposed LC model, we have encountered several challenges and limitations. The complexity of integrating multiple spline functions is one of the significant challenges. We fine-tuned and validated the ensembled model in order to guarantee the overall performance of the proposed model. Incorporating LIME with the proposed model caused challenges in maintaining computational power and performance. Training and deploying the proposed model demands substantial computational resources. In order to minimize the computational power and maximize the model’s efficiency, we employed quantization and pruning strategies. However, the performance of the proposed framework may vary in other datasets. The shortcomings of the PET / CT images in capturing small lung nodules may influence the capability of the proposed framework.

Furthermore, the variability in the medical imaging data and patient demographics may influence the model’s performance. We generalized the proposed model in a dataset covering a diverse population. However, generalizing the model in additional datasets may enhance the model’s efficacy. Data acquisition is one of the limitations due to privacy concerns and the sensitive nature of data. This may limit the proposed model’s capability across multiple clinical settings. Advanced feature extraction and fusion techniques can be used to enhance the performance of the proposed LC detection model.

## 6. Conclusion

The study highlights the potential of the feature fusion technique and ensemble learning-based KANs model in enhancing LC detection using PET / CT images. It leverages the strength of MobileNet V3 and LeViT feature extraction in identifying crucial LC features. Integrating multiple spline functions yielded a significant improvement in classifying the extracted features. The proposed study outperformed the traditional diagnostic approaches by utilizing PET / CT images and advanced DL techniques. The accuracy achieved was 99.0%, with considerable parameters of 19M and FLOPs of 27G, which underscores the effectiveness of the proposed model. The superior performance demonstrates the ability of the recommended model to detect LC in a resource-constrained environment. Integrating LIME with the proposed model offered the interpretability of the outcomes, leading to better management of LC diagnosis. The proposed study outlined the importance of ensemble learning in medical image analysis, paving the way for developing artificial intelligence applications across different types of cancer. The proposed study demands substantial generalization across diverse populations in order to enhance its effectiveness. The deployment of the proposed model requires expertise to fine-tune the performance in real-time settings. In the future, advanced feature extraction and fusion techniques will be essential to improve the generalization of the proposed model.

## References

[1] Kim Junghyun, and Kwan Hyoung Kim. "Role of chest radiographs in early lung cancer detection." Translational lung cancer research 9, no. 3 (2020): 522. doi: 10.21037/tlcr.2020.04.02 32676316 PMC7354112

[2] Jazieh Abdul Rahman, Algwaiz Ghada, Alshehri Salem M., and Alkattan Khaled. "Lung Cancer in Saudi Arabia." Journal of Thoracic Oncology 14, no. 6 (2019): 957–962. 31122559 10.1016/j.jtho.2019.01.023

[3] Jazieh Abdul Rahman, Majed AlGhamdi Sarah AlGhanem, Mohammed AlGarni Khaled AlKattan, Mashael AlRujaib, et al. "Saudi lung cancer prevention and screening guidelines." Annals of Thoracic Medicine 13, no. 4 (2018): 198–204. doi: 10.4103/atm.ATM_147_18 30416590 PMC6196665

[4] Candemir Sema, and Antani Sameer. "A review on lung boundary detection in chest X-rays." International journal of computer assisted radiology and surgery 14 (2019): 563–576. doi: 10.1007/s11548-019-01917-1 30730032 PMC6420899

[5] Xie Yutong, Xia Yong, Zhang Jianpeng, Song Yang, Feng Dagan, Fulham Michael, et al. "Knowledge-based collaborative deep learning for benign-malignant lung nodule classification on chest CT." IEEE transactions on medical imaging 38, no. 4 (2018): 991–1004. doi: 10.1109/TMI.2018.2876510 30334786

[6] Lee Sang Min, Joon Beom Seo Jihye Yun, Cho Young-Hoon, Jens Vogel-Claussen Mark L. Schiebler, et al. "Deep learning applications in chest radiography and computed tomography: current state of the art." Journal of thoracic imaging 34, no. 2 (2019): 75–85. doi: 10.1097/RTI.0000000000000387 30802231

[7] Asha, V., and K. Bhavanishankar. "Lung Cancer Detection using CT Scans: Image Processing through Deep Learning-A Review." In 2023 8th International Conference on Communication and Electronics Systems (ICCES), pp. 1203–1213. IEEE, 2023.

[8] Shah Hussain, Mubeen Iqra, Ullah Niamat, Shah Syed Shahab Ud Din, Khan Bakhtawar Abduljalil, Zahoor Muhammad, et al. "Modern diagnostic imaging technique applications and risk factors in the medical field: a review." BioMed research international 2022, no. 1 (2022): 5164970. doi: 10.1155/2022/5164970 35707373 PMC9192206

[9] Azam Kazi Sultana Farhana, Ryabchykov Oleg, and Bocklitz Thomas. "A review on data fusion of multidimensional medical and biomedical data." Molecules 27, no. 21 (2022): 7448. doi: 10.3390/molecules27217448 36364272 PMC9655963

[10] Saba Tanzila. "Recent advancement in cancer detection using machine learning: Systematic survey of decades, comparisons and challenges." Journal of infection and public health 13, no. 9 (2020): 1274–1289. doi: 10.1016/j.jiph.2020.06.033 32758393

[11] Jiang Huiyan, Diao Zhaoshuo, Shi Tianyu, Zhou Yang, Wang Feiyu, Hu Wenrui, et al. "A review of deep learning-based multiple-lesion recognition from medical images: classification, detection and segmentation." Computers in Biology and Medicine 157 (2023): 106726. doi: 10.1016/j.compbiomed.2023.106726 36924732

[12] Abhir Bhandary, Prabhu G. Ananth, Rajinikanth Venkatesan, Thanaraj K. Palani, Satapathy Suresh Chandra, Robbins David E., et al. "Deep-learning framework to detect lung abnormality–A study with chest X-Ray and lung CT scan images." Pattern Recognition Letters 129 (2020): 271–278.

[13] Han Yong, Ma Yuan, Wu Zhiyuan, Zhang Feng, Zheng Deqiang, Liu Xiangtong, et al. "Histologic subtype classification of non-small cell lung cancer using PET/CT images." European journal of nuclear medicine and molecular imaging 48 (2021): 350–360. doi: 10.1007/s00259-020-04771-5 32776232

[14] Yuan Lili, An Lin, Zhu Yandong, Duan Chongling, Kong Weixiang, Jiang Pei, et al. "Machine Learning in Diagnosis and Prognosis of Lung Cancer by PET-CT." Cancer Management and Research (2024): 361–375. doi: 10.2147/CMAR.S451871 38699652 PMC11063459

[15] Kerhet A., Small C., Quon H., Riauka T., Schrader L., Greiner R., et al. "Application of machine learning methodology for PET-based definition of lung cancer." Current oncology 17, no. 1 (2010): 41–47. doi: 10.3747/co.v17i1.394 20179802 PMC2826776

[16] Teramoto Atsushi, Yamada Ayumi, Tsukamoto Tetsuya, Imaizumi Kazuyoshi, Toyama Hiroshi, Saito Kuniaki, et al. "Decision support system for lung cancer using PET/CT and microscopic images." Deep Learning in Medical Image Analysis: Challenges and Applications (2020): 73–94. doi: 10.1007/978-3-030-33128-3_5 32030664

[17] Sadad Tariq, Rehman Amjad, Hussain Ayyaz, Abbasi Aaqif A., and Khan Muhammad Qasim. "A review on multi-organ cancer detection using advanced machine learning techniques." Current medical imaging 17, no. 6 (2021): 686–694. doi: 10.2174/1573405616666201217112521 33334293

[18] Raza Rehan, Zulfiqar Fatima, Muhammad Owais Khan Muhammad Arif, Alvi Atif, Iftikhar Muhammad Aksam, et al. "Lung-EffNet: Lung cancer classification using EfficientNet from CT-scan images." Engineering Applications of Artificial Intelligence 126 (2023): 106902.

[19] Kumar Ashnil, Fulham Michael, Feng Dagan, and Kim Jinman. "Co-learning feature fusion maps from PET-CT images of lung cancer." IEEE Transactions on Medical Imaging 39, no. 1 (2019): 204–217. doi: 10.1109/TMI.2019.2923601 31217099

[20] Guo, Ning, Ruoh-Fang Yen, Georges El Fakhri, and Quanzheng Li. "SVM based lung cancer diagnosis using multiple image features in PET/CT." In 2015 IEEE Nuclear Science Symposium and Medical Imaging Conference (NSS/MIC), pp. 1–4. IEEE, 2015.

[21] Oh Seungwon, Im Jaena, Kang Sae-Ryung, Oh In-Jae, and Kim Min-Soo. "PET-based deep-learning model for predicting prognosis of patients with non-small cell lung cancer." IEEE Access 9 (2021): 138753–138761.

[22] Rayed Md Eshmam, SM Sajibul Islam Sadia Islam Niha, Jamin Rahman Jim Md Mohsin Kabir, and Mridha M. F. "Deep learning for medical image segmentation: State-of-the-art advancements and challenges." Informatics in Medicine Unlocked (2024): 101504.

[23] Barbouchi Khalil, Dhekra El Hamdi Ines Elouedi, Takwa Ben Aïcha Afef Kacem Echi, and Slim Ihsen. "A transformer‐based deep neural network for detection and classification of lung cancer via PET/CT images." International Journal of Imaging Systems and Technology 33, no. 4 (2023): 1383–1395.

[24] Protonotarios Nicholas E., Katsamenis Iason, Sykiotis Stavros, Dikaios Nikolaos, Kastis George A., Chatziioannou Sofia N., et al. "A few-shot U-Net deep learning model for lung cancer lesion segmentation via PET/CT imaging." Biomedical Physics & Engineering Express 8, no. 2 (2022): 025019. doi: 10.1088/2057-1976/ac53bd 35144242

[25] Park Yong-Jin, Choi Dongmin, Choi Joon Young, and Hyun Seung Hyup. "Performance evaluation of a deep learning system for differential diagnosis of lung cancer with conventional CT and FDG PET/CT using transfer learning and metadata." Clinical Nuclear Medicine 46, no. 8 (2021): 635–640. doi: 10.1097/RLU.0000000000003661 33883488

[26] Sepehri Shima, Tankyevych Olena, Upadhaya Taman, Visvikis Dimitris, Hatt Mathieu, and Le Rest Catherine Cheze. "Comparison and fusion of machine learning algorithms for prospective validation of PET/CT radiomic features prognostic value in stage II-III non-small cell lung cancer." Diagnostics 11, no. 4 (2021): 675. doi: 10.3390/diagnostics11040675 33918681 PMC8069690

[27] Chen Song, Han Xiangjun, Tian Guangwei, Cao Yu, Zheng Xuting, Li Xuena, and Li Yaming. "Using stacked deep learning models based on PET/CT images and clinical data to predict EGFR mutations in lung cancer." Frontiers in Medicine 9 (2022): 1041034. doi: 10.3389/fmed.2022.1041034 36300191 PMC9588917

[28] Huang Brian, Sollee John, Luo Yong-Heng, Reddy Ashwin, Zhong Zhusi, Wu Jing, et al. "Prediction of lung malignancy progression and survival with machine learning based on pre-treatment FDG-PET/CT." EBioMedicine 82 (2022). doi: 10.1016/j.ebiom.2022.104127 35810561 PMC9278031

[29] Sengodan Prabaharan, Srinivasan Karthik, Pichamuthu Rajaram, and Matheswaran Saravanan. "Early detection and classification of malignant lung nodules from CT images: An optimal ensemble learning." Expert Systems with Applications 229 (2023): 120361.

[30] Onozato Yuki, Iwata Takekazu, Uematsu Yasufumi, Shimizu Daiki, Yamamoto Takayoshi, Matsui Yukiko, et al. "Predicting pathological highly invasive lung cancer from preoperative [18F] FDG PET/CT with multiple machine learning models." European journal of nuclear medicine and molecular imaging 50, no. 3 (2023): 715–726. doi: 10.1007/s00259-022-06038-7 36385219 PMC9852187

[31] Sultana, Zakia, Md Foysal, Soyabul Islam, and Abir Al Foysal. "Lung Cancer Detection and Classification from Chest CT Images Using an Ensemble Deep Learning Approach." In 2024 6th International Conference on Electrical Engineering and Information & Communication Technology (ICEEICT), pp. 364–369. IEEE, 2024.

[32] Li P., Wang S., Li T., Lu J., HuangFu Y., & Wang D. (2020). A Large-Scale CT and PET/CT Dataset for Lung Cancer Diagnosis (Lung-PET-CT-Dx) [Dataset]. The Cancer Imaging Archive. doi: 10.7937/TCIA.2020.NNC2-0461

[33] Liu Z, Wang Y, Vaidya S, Ruehle F, Halverson J, Soljačić M, et al: Kolmogorov-arnold networks. arXiv preprint arXiv:2404.19756. 2024 Apr 30.

